# AFM1 Detection in Milk by Fab’ Functionalized Si_3_N_4_ Asymmetric Mach–Zehnder Interferometric Biosensors

**DOI:** 10.3390/toxins11070409

**Published:** 2019-07-14

**Authors:** Tatevik Chalyan, Cristina Potrich, Erik Schreuder, Floris Falke, Laura Pasquardini, Cecilia Pederzolli, Rene Heideman, Lorenzo Pavesi

**Affiliations:** 1 Nanoscience Laboratory, Department of Physics, University of Trento, 38123 Trento, Italy; 2 LaBSSAH, Fondazione Bruno Kessler, 38123 Trento, Italy; 3 CNR-Consiglio Nazionale delle Ricerche, Istituto di Biofisica, 38123 Trento, Italy; 4 LioniX International BV, 7521 AN Enschede, The Netherlands

**Keywords:** lab-on-chip, optical biosensors, Fab’, Aflatoxin M1, asymmetric Mach–Zehnder interferometer, limit of detection, affinity

## Abstract

Aflatoxins (AF) are naturally occurring mycotoxins, produced by many species of Aspergillus. Among aflatoxins, Aflatoxin M1 (AFM1) is one of the most frequent and dangerous for human health. The acceptable maximum level of AFM1 in milk according to EU regulation is 50 ppt, equivalent to 152 pM, and 25 ppt, equivalent to 76 pM, for adults and infants, respectively. Here, we study a photonic biosensor based on Si${}_{3}$N${}_{4}$ asymmetric Mach–Zehnder Interferometers (aMZI) functionalized with Fab’ for AFM1 detection in milk samples (eluates). The minimum concentration of AFM1 detected by our aMZI sensors is 48 pM (16.8 pg/mL) in purified and concentrated milk samples. Moreover, the real-time detection of the ligand-analyte binding enables the study of the kinetics of the reaction. We measured the kinetic rate constants of the Fab’-AFM1 interaction.

## 1. Introduction

Contamination of food and agricultural products by various types of toxigenic molds (fungi) is a serious and global problem in the increasing number of countries. Fungal toxins have been detected in various food commodities and have been recognized to be one of the most dangerous contaminants that affects human and animal health [1]. In particular, mycotoxins produced by several species of fungi are naturally present in nuts, grains, maize, rice, soya [2]. Out of different categories of mycotoxins, aflatoxins produced by toxigenic strains of the fungi *Aspergillus flavus* and *Aspergillus parasiticus* are recognized to be the most toxic/carcinogenic compounds [3,4]. Aflatoxins found in feed are known as aflatoxin B1, B2, G1, and G2. In milk aflatoxin is present as AFB1 metabolite known as Aflatoxin M1 (AFM1). The International Agency for Research on Cancer (IARC) has categorized AFM1 as 2B human carcinogen [5]. Since most of the human species, as well as animals, particularly the nurturing ones, are dependent from milk as a part of complete basal nutrition, AFM1 contamination in milk and its products is of extreme importance and is a serious problem. The European Commission (EC) regulation No. 1881/2006 specifies the maximum level of AFM1 contamination in milk to 50 ppt (50 pg/mL) for adults, and to 25 ppt (25 pg/mL) for infant formulae. However, a long-term consumption of AFM1 at the low levels (ppb) may lead to the development of hepatocellular cancer [6]. In addition, these mycotoxins are thermostable and very resistant to degradation, even after chemical treatments [7]. Therefore, milk contaminated by AFM1 has to be eliminated before entering to the human nutrition.

Methods for fast and effective detection of AFM1 are indeed crucial. However, they have a non-negligible economic impact, being unavailable especially in the poor developing countries, where the level of milk contamination is high. Considering the sensitivity, reproducibility, specificity and cost per unit of test, standard methods such as thin layer chromatography (TLC) [8], High Performance Liquid Chromatography (HPLC) [9,10], immunoassays such as enzyme-linked immunosorbent assay (ELISA) [11,12] have been traditionally identified as more convenient tools for the detection of aflatoxins. These methods are based on fluorescence or electrochemical principles. On the other hand, alternative sensing devices based on optics are getting more and more appealing due to their speed, low cost and performances. Specifically, surface plasmon resonance (SPR) biosensors today are the most popular and commercialized optical biosensors [13]. They are widly used for affinity analyses [14]. They are competitive with HPLC or ELISA. Table 1 shows the comparison of the results from the known AFM1 detection systems with the results reported in this work.

Since 1990, the development of the integrated photonics-based optical biosensors gained momentum as a result of the optimization of the microfabrication technologies. Thereafter, label-free optical biosensors have become one of the most attractive biosensing devices thanks to their selectivity and high sensitivity [22]. Moreover, the easy fabrication with standard microelectronic/micromachining processes guarantees a low cost for mass production. These types of devices satisfy the requirements of the portability and, thus, they free the biosensors from the laboratory settings. This is a result of the integration with microfluidics which allows the realization of lab-on-chip devices [23].

Lab-on-a-chip devices got a further impulse after the development of silicon photonics. Silicon photonics takes advantage of the several favorable optical properties of silicon, such as the high refractive index that enables the realization of small optical components or the easy of manufacturing silicon by using complementary metal oxide semiconductor (CMOS) technology [24]. In silicon photonics based biosensors, micro-ring resonators (MRR) based on whispering gallery mode (WGM) cavities and interferometers, such as the Mach–Zehnder interferometer (MZI), are the preferred geometries. The use of a small micro-resonator for biosensing applications made its first appearance only in recent years [25,26]. Different proteins, down to a single molecule level [27], have been detected. In 1993 Heideman et al. [28], demonstrated a biosensor based on MZI using a planar optical waveguide as the sensing element. Biosensor, functionalized with antibodies, successfully detected human chorionic gonadotropin (hCG). Since then many works have been reported on integrated MZI biosensors [29,30,31,32].

In search of an economic, portable device to detect AFM1 in milk in less than 1 h and to reach a limit of detection of AFM1 comparable with available commercial systems, the European project SYMPHONY [33] (grant number 610580) has developed a system, based on an integrated photonic sensor, interfaced with a complex microfluidic stage to purify and concentrate the milk samples. The photonic sensor is based on an array of asymmetric Mach–Zehnder interferometers (aMZI). The sensing is performed by measuring the phase shift of the output signal, caused by the binding of the analyte on the functionalized aMZI surface. The binding causes changes on the effective refractive index, n${}_{eff}$, of the optical mode, confined in the waveguide. The change on n${}_{eff}$ is converted to the phase shift between the signal from the sensing and the reference arms of the interferometer. In order to have a specific detection of AFM1, a functionalization process, based on antigen-binding fragments (Fab’) is applied to the surface of the sensing arm of an aMZI. The main advantage of using Fab’, with respect to whole antibodies, is the possibility to obtain a higher surface density of the Fab’ fragments which yields a higher biosensor sensitivity as well as a lower limit of detection (LOD${}_{AFM1}$) of AFM1 [34].

In our previous works, we have reported an example of a complete measurement cycle for AFM1 detection in MES buffer as well as AFM1 specificity measurements in comparison with ochratoxin A (OTA) mycotoxin in MES buffer [35]. It has been demonstrated that for a solution which contains 5 nM of AFM1 injected in the Fab’ functionalized sensor the spectral shift is much higher than if we inject a solution with 100 nM of OTA.

Here we focus on AFM1 monitoring at different concentrations in milk samples. First, we report the optical characterization of the sensors such as the bulk sensitivity, S${}_{b}$, and the LOD. Then, we perform biosensing measurements in milk samples. Finally, we measure the AFM1-Fab’ binding affinity and the dissociation constant in milk. The lowest detectable concentration of AFM1 in milk by our sensor is 48 pM, lower than the EU regulations request.

## 2. Results

### 2.1. Experimental Setup

**Figure 1:**
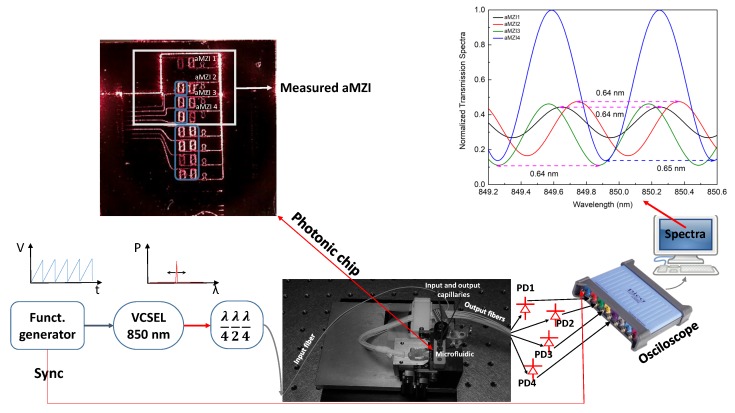
(**top left**) Image of the chip with illuminated Mach–Zehnder Interferometers (aMZI). (**top right**) Normalized transmission spectra of four aMZI. (**bottom**) Schematic of the experimental setup.

An image of the sensor chip is reported in Figure 1. The devices were fabricated by the single-stripe TriPlex technology [36]. The details of the sensor fabrication process are given in [35].

The sensor design had eight aMZI in a single chip. Each aMZI had two arms realized by spiral waveguides to keep the footprint small. The length of the waveguide in each arm was 6.25 mm. There was a small additional length in one of the two arms, named the reference arm, to obtain an asymmetry. This additional length determines the period of the interference fringes when the input signal wavelength is scanned, i.e., the free spectral range (FSR) of the aMZI. The FSR is set such that it matches the spectral tuning range of the laser source, which was a commercial vertical-cavity surface-emitting laser (VCSEL) emitting at 850 nm. An input waveguide (bright spot on the right in Figure 1) feds two one-to-four channel splitters which distribute the input signal to two groups of four aMZI. In this work, we only use the top set formed by four aMZIs, three of which have an exposed arm. By this we mean that the cladding on top of them is removed to form a sensing window. The sensing window allows the interaction between the evanescent field of the light propagating in the waveguide and the liquid sample of interest. To open the sensing window, the top cladding is locally removed by a photolithography step and BHF wet etch, down to the Si${}_{3}$N${}_{4}$ layer. One aMZI (the top one in Figure 1) is left covered to save as a reference for the temperature or laser fluctuations. The signals from the aMZI are then individually sent to the outputs.

In fact, the sensor device was mounted in a miniaturized alignment stage [35] completed with a microfluidic chamber with two inlets. These are connected to a VICI M6 liquid handling pump with capillaries with a diameter of 150 μm which are used for the continuous flow during the sensing measurements. A single mode fiber at 850 nm connects the mentioned light source (VCSEL) and the chip. The polarization of the input light signal is controlled by a two-paddle polarization stages. For the detection, we connected the output fibers to four Si transimpedance amplified photodetectors interfaced to a PicoScope 4824 (an eight channel USB oscilloscope). Wavelength scanning was achieved by current tuning the VCSEL with a periodic current ramp, which also triggers the time scan of the oscilloscope. In this way, a wavelength scan of the four aMZI is performed and the output signals are recorded by the oscilloscope and transferred to a control computer. Figure 1, top right, reports an example of the recorded waveforms from the four aMZI on the same photonic chip. Any variation of the effective indices of the mode travelling in the sensing arm causes a phase shift of the waveforms of the output signals. Therefore, by real-time analysis of the waveforms, a live recording of the phase shifts of the signal light propagating in the four aMZI can be achieved with a VCSEL modulation frequency of 20 Hz, and a data acquisition of 50,000 points per spectrum. The transmission signals of all devices were normalized to the maximum signal of the reference aMZI. Apart from variation of the intensity and relative phase, the FSR of the three aMZI with the sensing arm are the same and equal to 0.64 nm. The FSR of the reference aMZI is slightly different as a result of the presence of the cladding. Knowing the FSR, we can calculate the wavelength shift from the phase shift, considering that 1 rad ≅$\frac{FSR}{2\pi }$ = 0.1014 nm. In order to compare results of various sensing methods, it was convenient to report the sensor response in nm and not in radians. To check the repeatability of the sensor production, we characterized more than 60 sensors.

### 2.2. Surface Preparation

Fab’ were prepared starting from rabbit anti-afltoxin M1 antiserum (Tecna). The functionalization is based on a mixed self assembled monolayer (SAM), a well known method utilized for the covalent immobilization of antibodies [37]. Polyclonal antibodies (IgG) were firstly purified from antiserum components with Amicon Ultra-0.5 10 kDa centrifugal filters (Millipore) to change to the suitable sample buffer (10 mM phosphate buffer, 10 mM ethylenediaminetetraacetic acid (EDTA) pH 7). IgG were then mixed with immobilized papain (ThermoScientific) pre-activated with cysteine-HCl, following manufacturer’s instructions. The digestion of IgG with papain was performed at 37 ${}^{\circ }$C for 6 h. At the end of digestion, three volumes of 10 mM Tris/HCl buffer pH 7.5 (with respect to the starting antibody volume) were added before spinning the reacted antibodies. The obtained supernatant contained the Fab’ fragments, the undigested IgG and Fc fragments, which were separated by ion exchange chromatography (Vivapure D Mini, Sartorius). The resulting flow through fraction containing the Fab’ fragments was quantified by spectrophotometry (Nanodrop ND-1000 spectrophotometer, Nanodrop Tecnologies, Wilmington, DE, USA) and the quality of Fab was verified by SDS-PAGE (an example is reported in Figure S1).

**Figure 2:**
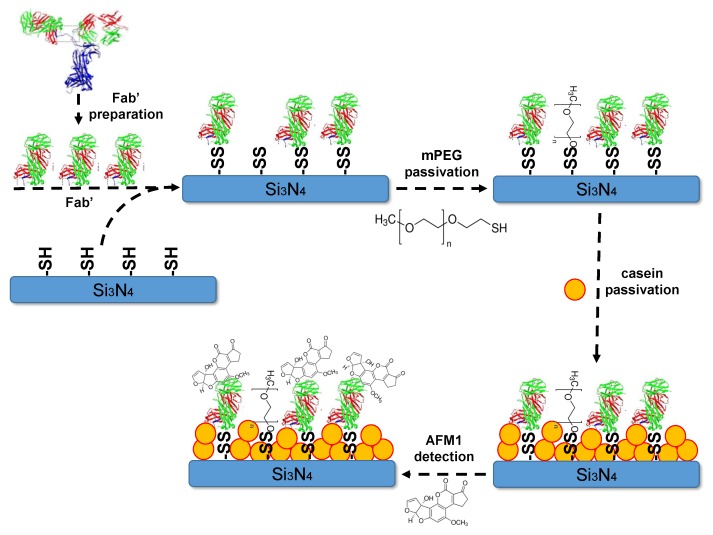
Schematics of the surface functionalization principle. Note, that the molecule sizes are not scaled and are not corresponding to the real proportions.

The desired amount of Fab’ was reduced for 2 h with 10 mM DTT just before use. The excess of DTT is then removed with Amicon Ultra-0.5 10 kDa centrifugal filters (Millipore). A general scheme of the surface functionalization is reported in Figure 2.

The Fab’ were immobilized on the Si${}_{3}$N${}_{4}$ surface of aMZI adapting the protocol described in Yoshimoto et al. for gold surfaces [38], but further characterizations were performed as reported in the Supplementary Material (Figures S2 and S3). Immobilization protocol for Si${}_{3}$N${}_{4}$ surface was described in the previous work of our group [39]. An additional passivation step was, indeed, carried out. An optimization of the surface passivation has been already reported [35]. Since in the milk samples there are casein molecules, non-specific adsorption of casein on the Si${}_{3}$N${}_{4}$ surface causes a significant fake signal parallel to the one from the Fab’-AFM1 specific binding. Thus, an additional step of casein passivation was carried out. Surfaces were passivated with 0.1 mg/mL of casein for 30 min then extensively rinsed in sample buffer (by using a becker with 150 mL of buffer), moved to a clean well of a 24-wells plate, covered with buffer and kept wet until used. In parallel with aMZI surfaces, Si${}_{3}$N${}_{4}$ flat surfaces are functionalized and used to check if the functionalization protocol was correct. At the end of functionalization, flat surfaces were incubated with AFM1-Horseradish Peroxidase (HRP) conjugate (part of Aflatoxin M1 ELISA kit I’screen, Tecna) for 1 h at room temperature on an orbital shaker at 60 rpm. After several washes with buffer (50 mM MES pH 6.6), these surfaces were incubated with the developer solutions (SuperSignal${}^{\mathrm{TM}}$ ELISA Femto Substrate, ThermoScientific) and measured with a ChemiDoc${}^{\mathrm{TM}}$ imaging system (BioRad, Figure S4).

### 2.3. Theoretical Model

The time evolution of the phase shift (sensorgrams) when the toxin is added to the sample follows the molecular binding events. Let us assume that this occurs via a simple 1:1 interaction (i.e., we assume that the ligand has only one binding site). Therefore, the ligand L and the target molecule A bind reversibly in solution to form a binary complex AL via

(1) $$L+A\left[k_{off}\right]k_{on}\mathrm{AL},$$

where k${}_{on}$ is the second-order rate constant for complex association and k${}_{off}$ is the first-order rate constant for complex dissociation. The rate of complex formation (whose concentration is [AL]) depends on the free concentration of A and L ([A] and [L] correspondingly) and on the stability of the complex. Since our sensor functionalized by Fab’ for AFM1 detection works by this principle [35], the characteristics of the molecular reaction Equation (1) can be extracted from a fit of the sensorgrams with a kinetic model. For various toxin concentrations, *C*, the following fit function is used [40]:

(2) $$R_{A}\left(t\right)=R_{eq}\left[1-\exp\left(-k_{obs}t\right)\right]\;,$$

where R${}_{A}$(*t*) is the sensor response (phase shift) at time *t*, R${}_{eq}$ is the signal level at equilibrium and k${}_{obs}$ is the experimentally determined value of the pseudo-first-order rate constant to approach the equilibrium. The latter is given as:

(3) $$k_{obs}=k_{on}C+k_{off}.$$

The linear fit of k${}_{obs}$ vs. C yields k${}_{on}$ and k${}_{off}$. Knowing k${}_{on}$ and k${}_{off}$, we can compute the complex equilibrium dissociation constant K${}_{D}$ or the molecular affinity K${}_{A}$:

(4) $$K_{D}=\frac{k_{off}}{k_{on}}=\frac{1}{K_{A}}\;.$$

## 3. Discussion

### 3.1. Bulk Sensitivity and Limit of Detection

We test the performances of our photonic sensors, by characterizing simultaneously the volume (bulk) Sensitivity (S${}_{b}$) of the three uncovered aMZIs. To calculate this parameter, a real-time monitoring of the phase shift of the aMZI is conducted, when glucose or salt (NaCl) water solutions of various concentrations flow on the sensor. Flowing pure deionized water provides the reference and the baseline. Let us define, the bulk sensitivity of the aMZIs as:

(5) $$S_{b}=\frac{\partial \phi_{0}}{\partial n_{s}}\;,$$

where $\phi_{0}$ is the phase and n${}_{s}$ is the cladding refractive index.

**Figure 3:**
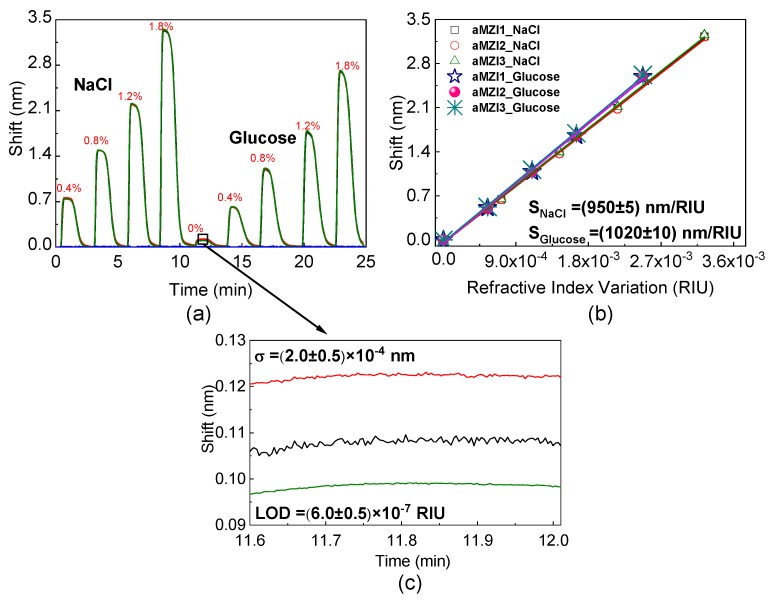
(**a**) Sensorgrams of four aMZI on the same chip for variation in the cladding refractive index, obtained by injections of glucose and salt water solutions at different concentrations (glucose and NaCl concentrations are in %*w*/*v* labelled on the plot). (**b**) Wavelength shift linear fit where the slope is the sensitivity. (**c**) The shift taken in 24 s corresponding to the time interval 11.6 ÷ 12.0 min in the sensorgram. This interval is marked inside the black square.

Figure 3a shows the wavelength shifts in nm when the cladding refractive index is changed. The plot refers to the simultaneous measure of the four aMZI. Note, that the results of the measurements reported in this figure correspond to ona photonic chip. The injection of the glucose or salt solutions causes a significant shift, which is similar for the three uncovered aMZIs on the chip. Since aMZI4 is covered, the change of the flowing liquid do not lead to any wavelength shift. The 0% value on the plot refers to the injection of pure water from the same reservoir of the flowing glucose or salt solutions. The observed small shift is caused by the temperature difference between the solution injected from the valve and that flowing continuously inside the tubings. In the further analyses this shift is considered as a baseline.

Figure 3b shows the dependence of the shift versus the refractive index of the solution, i.e., the slope of this plot is the bulk sensitivity. The refractive index variation is given by refractive index units (RIU). We find a sensitivity of (950 ± 5) nm/RIU and (1020 ± 10) nm/RIU, respectively, for NaCl and glucose water solutions for all the three exposed sensors. The small difference between these sensitivities can be a result of the density difference. In fact, there are more molecules of salt than of glucose in a solution with the same concentration when we measure the concentration in percentage of weight per volume. The measurement of both the glucose and salt solutions shows that for non-biological solutions the sensitivity is almost the same.

The minimum concentration that can be distinguished with a certain confidence level defines the limit of detection (LOD) of the biosensor [41]. Knowing S${}_{b}$, LOD can be calculated as:

(6) $$\mathrm{LOD}=\frac{k\sigma }{S_{b}}\;,$$

where σ is the standard deviation of repeated measurements of blank solutions. The use of k = 3 sets the confidence level to 99.7%.

By measuring an average standard deviation of the signal σ = (2.0 ± 0.5) × 10${}^{-4}$ nm within a time interval of 24 s (see Figure 3c), we calculate a LOD $≅\left(6.0\pm 0.5\right)\times 10^{-7}$ RIU.

**Figure 4:**
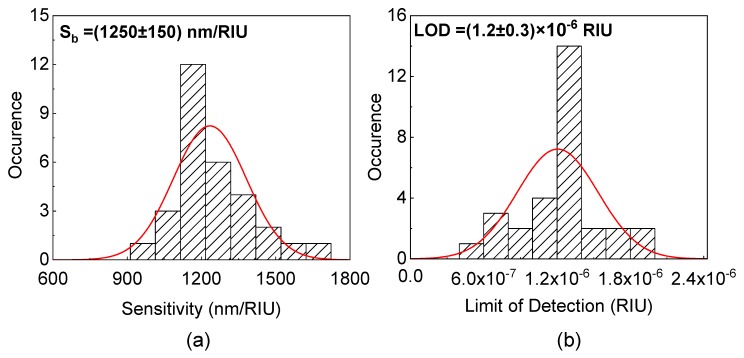
Histograms of the bulk sensitivity (S${}_{b}$) and of the limit of detection (LOD) for 60 aMZI chips: (**a**) The histogram for the bulk sensitivity obtained by volumetric sensing measurements. The mean value is (1250 ± 150) nm/RIU, the best measured sensitivity is (1600 ± 100) nm/RIU. Bin size is 100 nm/RIU. (**b**) The histogram for the LOD with 2 $\times 10^{-7}$ RIU bin size. The minimum LOD is (5.0 ± 1.0) ×$10^{-7}$ RIU, the mean value is LOD = $\left(1.2\pm 0.3\right)\times 10^{-6}$ RIU.

In order to verify the reproducibility of the sensor fabrication, we repeat the volumetric sensing measurements for more than 60 different sensors. The sensitivity and LOD histograms are reported in Figure 4. An ≈ 10% spread for the mean value of the sensitivity is observed which is an indication of the repeatability of the sensor fabrication and testing. A mean value for $S_{b}\approx \left(1250\pm 150\right)$ nm/RIU is achieved, while a mean value of LOD ≈(1.2±0.3)×$10^{-6}$ RIU is calculated. These values are comparable with other sensors reported in the literature [35].

### 3.2. AFM1 Detection

Sensing experiments are performed on filtered and concentrated samples where the toxins are concentrated by a factor of 20 in the eluate with the concentration module of the *SYMPHONY* setup. The AFM1 concentrations in the samples are first determined with the ELISA assay. For each concentration, we use freshly functionalized sensor. This is done in order to avoid surface functionality degradation after the regeneration process.

**Figure 5:**
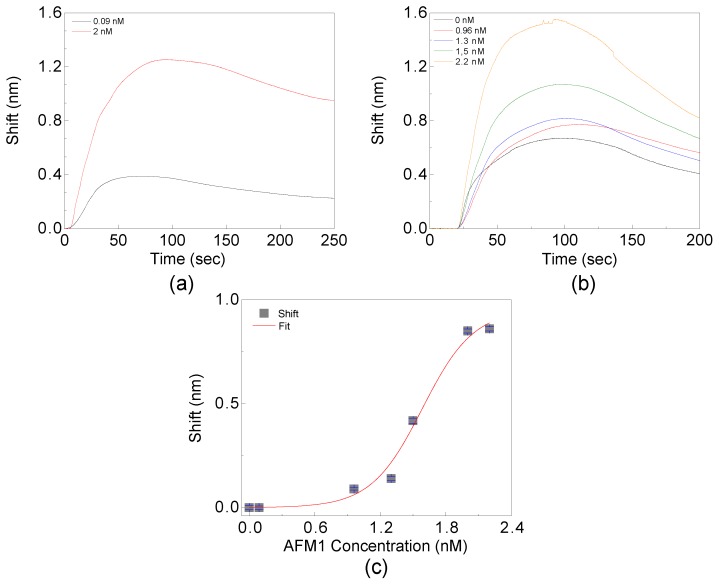
Aflatoxin M1 (AFM1) detection in the milk samples. Sensorgrams for concentrated milk samples with different AFM1 concentrations. (**a**) The first demonstration of AFM1 detection in milk. (**b**) The second set of tests of AFM1 detection. The black line is the response to the blank eluate. (**c**) The calibration function for the aMZI based sensor is extracted from the logistic fit of the shift values for different AFM1 concentrations (C).

For the first demonstration of AFM1 specific detection two eluates with 0.09 nM and 2 nM AFM1 concentrations, respectively (see Figure 5a) are used. A flow rate of 15 μL/min and an injected sample volume of 30 μL are used. Note that in the prepared samples still some proteins and, in particular, casein remain causing a non-specific signal. The non-specific adsorption of milk component even on passivated surfaces is a known phenomenon [21]. A clear difference between the measured signals is observed due to the different AFM1 concentrations. After getting confidence that the sensor distinguishes the AFM1 presence in the real milk samples, for the second set of the tests a wider variety of AFM1 concentrations is used. To achieve a faster detection, the flow rate is increased up to 20 μL/min. In this way, the measurement duration is reduced to 90 s. This time the first test of milk is performed on a sample free from AFM1 (blank sample) used as the reference for the sensor. Next, we inject milk samples containing 0.96 nM, 1.3 nM, 1.5 nM and 2.2 nM AFM1, respectively. We observe a wavelength shift which increases with the concentration (see Figure 5b).

In the second set of measurements, a higher signal for the blank sample than for the 0.09 nM AFM1 measurement of the first set is observed. Thus we infer that the 0.09 nM AFM1 concentration is not distinguishable for the sensor and that the signal for this concentration is due to the non-specific adsorption of other milk components. Likewise for the signal for the blank eluate, the signal is considered as a baseline with respect to the other measurements. Note, that the difference of non-specific signal levels in these two cases is caused by the different flow rates and the different buffers. In other words, eluates deriving from the concentrator module can vary in terms of milk components content and this variability is measured by the photonic sensor. Therefore, we set the LOD of the aMZI sensor to the AFM1 concentration of 0.96 nM. Taking into account the concentration factor of 20 times, this corresponds to an AFM1 concentration of 48 pM in the original milk sample. This estimate assumes that no AFM1 loss occurs in the concentration stage.

By referring the signals to the baselines and by plotting the dependence of the maximum shift at t = 90 s versus the AFM1 concentration, we can build the calibration curve of the sensor (see Figure 5c). In the figure, the error bars show the variation of the shift in a time range t = 90 ± 5 s. We fit the data with the following logistic function:

(7) $$R\left(C\right)=\frac{L}{\left(1+e^{-k(C-C_{0})}\right)}\;.$$

where *R* is the maximum shift and *C* is the AFM1 concentration, respectively.

From the fit we obtain the values for *L* = 0.95 ± 0.07; $C_{0}$ = 1.6 ± 0.06 and *k* = 4.3 ± 0.9 yielding to the following callibration function for the sensor:

(8) $$R\left(C\right)=\frac{1}{1.05(1+975e^{-4.3C})}\;.$$

**Figure 6:**
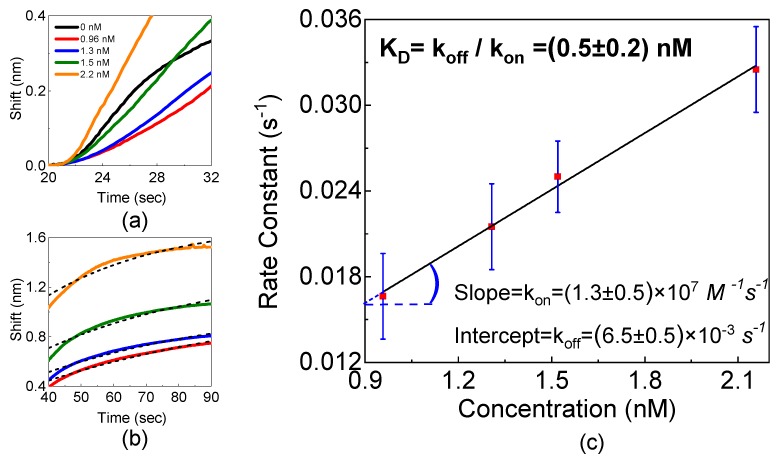
(**a**) The initial slopes of the sensor response for different concentrations of AFM1 in eluates. The black curve corresponds to the blank eluate. It shows a different behavior and a different kinetics supporting the fact that for the other curves the specific binding of AFM1 molecules is taking place. (**b**) The exponential fit (dashed lines) of the sensor response (continuous lines) in the 40 ÷ 90 s interval. (**c**) The dependence of the pseudo-first-order rate constant on the AFM1 concentrations. Slope and intercept of the linear fit correspond to k${}_{on}$ and k${}_{off}$.

A clear differences in the signal levels and kinetics (see Figure 6a) are observed between the reference sample and the ones with AFM1. This difference indicates that a specific interaction between AFM1 and Fab’ takes place, as expected. To further characterize the kinetics of the sensor response, we performed an exponential fit of the association region of the sensorgrams with Equation (2). From the fit of the curves in the 40 ÷ 90 s interval (see Figure 6b), where the association occurs, we obtain a linear dependence of the rate constants vs the AFM1 concentration (Figure 6c). From the linear fit, we extract k${}_{on}$ = (1.3 ± 0.5) × 10${}^{7}$ M${}^{-1}$s${}^{-1}$ and k${}_{off}$ = (6.5 ± 0.5) × 10${}^{-3}$s${}^{-1}$. From these values, we deduce K${}_{D}$ = (0.5 ± 0.2) × 10${}^{-10}$ M and K${}_{A}$ = (2 ± 1.5) × 10${}^{9}$ M${}^{-1}$. These values are comparable to the values for aptamer-AFM1 binding [42], while no data exists for the AFM1-Fab’ binding either in buffer or in real samples. The affinity value is comparable with the known affinity of this family of toxins which is in the range of 10${}^{9}$–10${}^{11}$ M${}^{-1}$ values [43].

## 4. Conclusions

In this work we demonstrated an asymmetric Mach–Zehnder Interferometric biosensor which, when functionalized with Fab’, is able to detect specifically AFM1 in concentrated and filtered milk samples down to ≈48 pM. This LOD is comparable with other state-of-the-art devices (see Table 1). The aMZI described here shows an improved response time compared to the ones reported in the literature [44]. In addition, with respect to SPR sensors, the aMZI biosensor is more compact, easy to operate and cheaper.

Owing to the setup capabilities the kinetic of the AFM1-Fab’ reaction has been characterized as well. Affinity constants of K${}_{A}$ = (2 ± 1.5) × 10${}^{9}$ M${}^{-1}$ in milk are measured which are comparable with the affinities of the same family of antibodies-AFM1 interaction.

This work opens new perspectives for integrated photonic biosensors as viable solutions for lab-on-chip devices for food safety analyses.

## 5. Materials and Methods

### Milk Sample Preparation

Fresh whole milk was spiked with AFM1 in order to prepare samples to be processed for the detection of AFM1 with the biosensor [33]. We collected 400 mL of milk in glass bottles pretreated with 3% BSA; then milk was spiked with different concentrations of AFM1 and incubated at 40 ${}^{\circ }$C for 1 h before placing at 4 ${}^{\circ }$C until used.

After defatting and pre-concentration/purification treatments, eluted fractions containing AFM1 (quantified with Aflatoxin M1 ELISA kit I’screen, Tecna) and control fractions prepared starting from milk without Aflatoxin, were tested with aMZI biosensors.

## Figures and Tables

**Table 1:** Comparison of various methods to detect Aflatoxin M1 (AFM1) in milk.

Method	LOD (ppt)	Detection Time (min)	Reference
TLC	5	4–5	[8]
HPLC	4.5	72	[9,10]
ELISA	4.3	3	[11,12]
ROSA	500	0.15	[15]
Bilayer Lipid Membranes	16	0.5	[16]
Microelectrodes Arrays	8	2	[17]
Electrochemical	10	0.5	[18]
Field Immunoassay	50	3	[19]
DNA-aptasensor	20	4	[20]
SPR	0.6	1	[13]
SPR with gold nanoparticles	18	1	[21]
aMZI	16.8	1.5	This work

## Supplementary Materials

The following are available online at https://www.mdpi.com/2072-6651/11/7/409/s1, Figure S1: SDS-PAGE of total digest before separation by ion exchange chromatography (lane 2) and after separation: flow through fraction (lane 3), wash fraction (lane 4), eluted fraction (lane 5). Lane 1 and 6: LMW-SDS markers (GE Healthcare Life Science). SDS-PAGE was performed using precast minigels with density gradients ranging 10–15% (GE Healthcare Life Science). A semiautomatic horizontal unit (PhastSystem by Pharmacia) was used. The gel was stained with Coomassie brilliant blue, Figure S2: Chemiluminescence signal of Fab’-functionalized surfaces kept at 4 C until measured. Error bars represent the standard deviations, Figure S3: Optimization of surface passivation with casein. Upper panel: PEG-treated surfaces, without Fab’. Lower panel: Surfaces functionalized with Fab’ and a first step of passivation with PEG. NP: non-passivated surface, Figure S4: Chemiluminescence signal of 3 Si3N4 flat surfaces functionalized with Fab’ (1–3) or with all steps of the functionalization protocol except Fab’ (4–6). Surfaces were inserted in wells of a 96 wells black microplate for handling. No signal is visible were Fab’ are missing (wells 4–6).

## Abbreviations

The following abbreviations are used in this manuscript:

AFM1	Aflatoxin M1
AFB1	Aflatoxin B1
aMZI	Asymmetric Mach–Zehnder interferometer
CMOS	Complementary metal oxide semiconductor
ELISA	Enzyme-linked immunosorbent assay
FSR	Free spectral range
HPLC	High performance liquid chromatography
hCG	Human chorionic Gonadotropin
HRP	Horseradish Peroxidase
LOD	Limit Of detection
MES	2-(*N*-Morpholino)EthaneSulfonic acid
MZI	Mach–Zehnder interferometer
MRR	Micro ring resonator
OTA	Ochratoxin A
PDMS	Polydimethylsiloxane
RIU	Refractive Index Unit
SPR	Surface plasmon resonance
TLC	Thin layer chromatography
VCSEL	Vertical-cavity surface-emitting laser
WGM	Whispering gallery mode
