# Supplementary material for: AFM1 Detection in Milk by Fab’ Functionalized Si3N4 Asymmetric Mach–Zehnder Interferometric Biosensors
Source: Toxins (Basel). 2019 Jul 14;11(7):409. doi: 10.3390/toxins11070409 (PMC6669449; doi:10.3390/toxins11070409)
Supplement: Supplementary file 1 [file toxins-11-00409-s001.pdf]

Supplementary Materials: AFM1 Detection in Milk by Fab′ Functionalized Si3N4 Asymmetric Mach-Zehnder Interferometric Biosensors

Tatevik Chalyan, Cristina Potrich, Erik Schreuder, Floris Falke, Laura Pasquardini, Cecilia Pederzolli, Rene Heideman and Lorenzo Pavesi

Control of Fab′ Preparation

The preparation of Fab′ is described in the Material and Methods Section of the manuscript.

After digestion, the supernatant containing the Fab′ fragments, the undigested IgG and Fc fragments are separated by ion exchange chromatography (Vivapure D Mini, Sartorius). The resulting flow through fraction containing the Fab′ fragments is quantified by spectrophotometry (Nanodrop ND-1000 spectrophotometer, Nanodrop Tecnologies, Wilmington, DE, USA) and the quality of Fab is verified by SDS-PAGE (**Error! Reference source not found.**1).

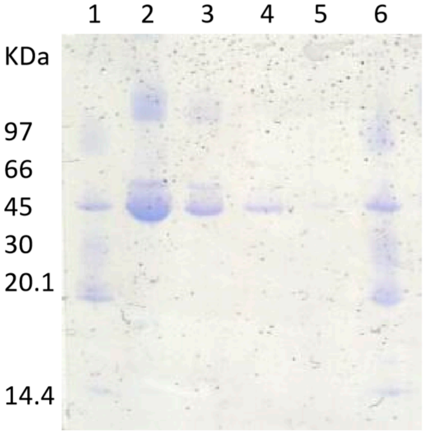

Figure S1. SDS-PAGE of total digest before separation by ion exchange chromatography (lane 2) and after separation: flow through fraction (lane 3), wash fraction (lane 4), eluted fraction (lane 5). Lane 1 and 6: LMW-SDS markers (GE Healthcare Life Science). SDS-PAGE was performed using precast minigels with density gradients ranging 10–15% (GE Healthcare Life Science). A semiautomatic horizontal unit (PhastSystem by Pharmacia) was used. The gel was stained with Coomassie brilliant blue.

Surface Characterization

The functionalization protocol was setup by testing its different steps, in particular the concentration of Fab′ to be incubated on the surface, the time of Fab′ incubation, aging of functionalized surfaces and passivation.

The optimization of Fab′-functionalized surface is described in detail in Chalyan et al. [1], where a titration of different concentration of Fab′ was tested. As explained in the paper, when 0.2 μM or more concentrated Fab′ are used, the surface is saturated and no more molecules are covalently bound. 0.33 μM Fab′ concentration was therefore selected as an excess of molecules for all experiments with the biosensor.

The time of incubation of Fab′ on the surfaces was also optimized by testing different conditions on flat surfaces [1]. The reaction of Fab′ on surface is almost immediate and the amount of bound molecules do not increase in time (up to 1 h). A short time was therefore selected (i.e., 2′) for the incubation of Fab′, before adding several passivating agents (i.e., mPEG-SH 5000, mPEG-SH 2000 and casein for a total time of 2 h).

In addition to concentration and time of incubation of Fab′, the aging of flat surfaces functionalized with Fab′ was tested. Surfaces were preserved at 4 °C in buffer (50 mM MES pH 6.6) until the chemiluminescence test was performed. At least 3 surfaces were tested for every condition. To detect Fab′ on surfaces, AFM1-HRP conjugate (part of Aflatoxin M1 ELISA kit I'screen, Tecna) was added for 1 h at room temperature on an orbital shaker at 60 rpm. After several washes with buffer (50 mM MES pH 6.6), these surfaces were incubated with the developer solutions (SuperSignalTM ELISA Femto Substrate, ThermoScientific, Waltham, MA, USA.) and measured with a ChemiDocTM Imaging System (BioRad, Hercules, CA, USA). A batch of surfaces was left at room temperature for 3 more h before measuring the chemiluminescence signal. **Error! Reference source not found.**2 shows that the functionalization is quite stable in time when the surfaces are maintained at 4 °C, while only when surfaces are left at room temperature for an overtime, a certain degree of variability among surfaces is measurable (i.e., standard deviation is higher).

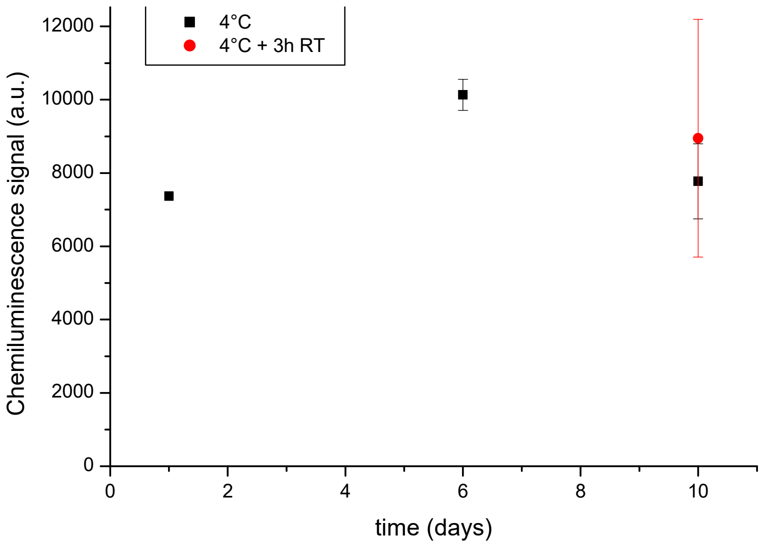

Figure S2. Chemiluminescence signal of Fab′-functionalized surfaces kept at 4 °C until measured. Error bars represent the standard deviations.

Surfaces functionalized with Fab′ were passivated both with mPEGs and with casein. The passivation step was optimized on flat surfaces, which were treated with increasing concentrations of casein (**Error! Reference source not found.**). Surfaces were incubated with AFM1-HRP and the resulting chemiluminescence signal was detected as explained above. A 0.1 mg/mL concentration of casein was selected as sufficient to completely suppressed the non-specific adsorption of aflatoxin on surfaces treated only with PEG (upper panel, **Error! Reference source not found.**3), without affecting the binding performances of Fab′ toward AFM1 (lower panel, **Error! Reference source not found.**).

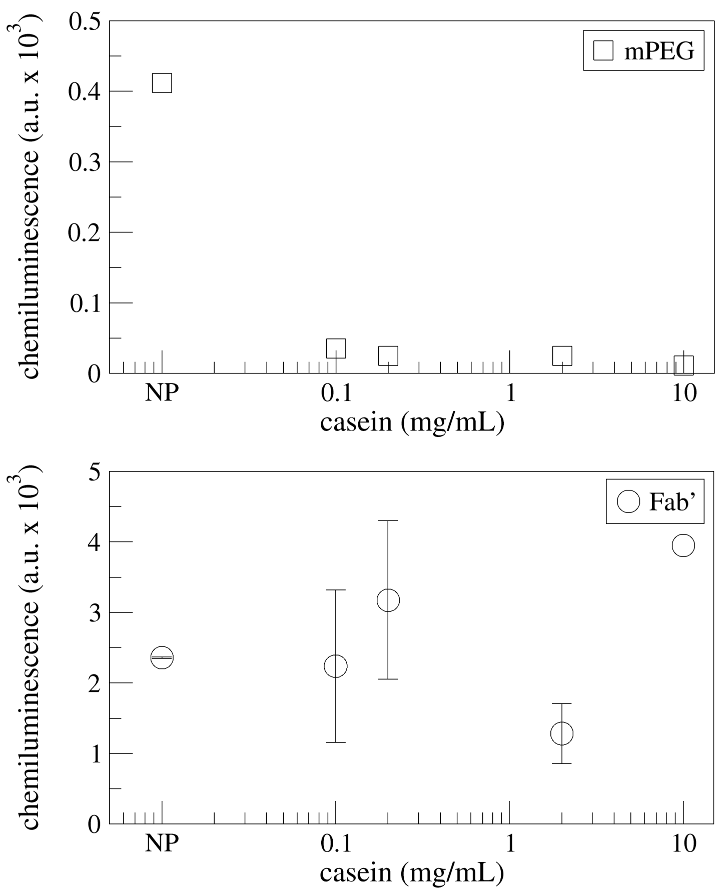

Figure S3. Optimization of surface passivation with casein. Upper panel: PEG-treated surfaces, without Fab′. Lower panel: Surfaces functionalized with Fab′ and a first step of passivation with PEG. NP: non-passivated

Control of Functionalized Surfaces

For each batch of Si3N4 asymmetric Mach-Zehnder Interferometers (aMZI) which was functionalized with Fab′, parallel Si3N4 flat surfaces were functionalized with the same protocol. At least 3 surfaces were functionalized each time with Fab′ and 3 without Fab′ but with all steps of functionalization. As explained above, AFM1-HRP conjugate (part of Aflatoxin M1 ELISA kit I'screen, Tecna S.r.l., Trieste, Italy) was incubated for 1 h at room temperature on an orbital shaker at 60 rpm. After several washes with buffer (50 mM MES pH 6.6), these surfaces are incubated with the developer solutions (SuperSignalTM ELISA Femto Substrate, ThermoScientific) and measured with a ChemiDocTM Imaging System (BioRad). An example of the result of this measure is shown in **Error! Reference source not found.**4.

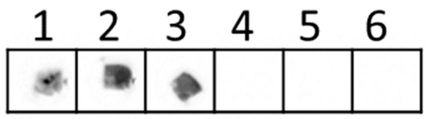

Figure S4. Chemiluminescence signal of 3 Si3N4 flat surfaces functionalized with Fab′ (1–3) or with all steps of the functionalization protocol except Fab′ (4–6). Surfaces were inserted in wells of a 96 wells black microplate for handling. No signal is visible were Fab′ are missing (wells 4–6).

Reference List

1. Chalyan, T.; Guider, R.; Pasquardini L.; Zanetti, M.; Falke, F.; Schreuder, E.; Heideman, R.G.; Pederzolli, C.; Pavesi L. Asymmetric Mach–Zehnder Interferometer Based Biosensors for Aflatoxin M1 Detection. *Biosensors* **2016**, *6*, 1–10.
